# Supplementary material for: Patient and family engagement in infection prevention in the context of the COVID-19 pandemic: defining a consensus framework using the Q methodology – NOSO-COVID study protocol
Source: BMJ Open. 2022 Jul 22;12(7):e056172. doi: 10.1136/bmjopen-2021-056172 (PMC9315236; doi:10.1136/bmjopen-2021-056172)
Supplement: Supplementary data [file bmjopen-2021-056172supp001.pdf]

Dimensions and items of the 40-items Q survey

Dimension	Item
**Infection prevention**	• P[1]/FM[2] should receive information on IPV[3] prior to being hospitalize/visit<br>• HP[4] should evaluate P/FM knowledge on IPV before giving them information<br>• HP should communicate information on IPV to P/FM prior to being hospitalize/visit
**Hand hygiene**	• HP are the only ones responsible for complying with HHM[5]<br>• P/FM have responsibilities for complying with HHM<br>• When visiting, FM should routinely practice HHM at key moments<br>• P should routinely practice HHM at key moment<br>• P/FM should ask HP to practice HHM if they notice it has not been done<br>• P/FM don't have the responsibility to check that HP practice HHM<br>• Information about HHM should be available on hospital units<br>• HP should regularly remind P/FM to practice HHM at key moments<br>• PP[6] should educate/encourage P/FM to practice HHM<br>• Hand washing/disinfection stations should be accessible to P/FM<br>• HP should check that P/FM practice HHM at key moments<br>• HP should encourage P/FM to ask HP to wash their hands if they notice it has not been done<br>• Reminders should be available to encourage P/FM to ask HP to wash their hands if they notice it has not been done
**Protective equipment**	• P (outside their room)/FM (entering the hospital) should wear a mask<br>• P (outside their room)/FM (entering the hospital) should not wear a mask<br>• P/FM should ask HP to wear their gloves/mask if they notice it has not been done<br>• P/Fm don't have the responsibility to check that healthcare professionals wear their gloves and mask<br>• Information about mask wearing should be available on hospital units<br>• HP should regularly remind P/FM to wear a mask<br>• PP should educate/encourage P/FM to wear a mask<br>• HP should check that P/FM wear a mask<br>• Masks should be available/accessible on the units for P/FM
**Disinfection**	• P/FM should have the responsibilities to disinfect equipment/immediate surroundings<br>• P/FM do not have the responsibility to disinfect the equipment/immediate surroundings<br>• Information about disinfection should be available to P/FM<br>• HP should regularly remind P/FM to disinfect equipment/immediate surroundings<br>• PP should educate/encourage P/FM to disinfect equipment/immediate surroundings<br>• Disinfecting wipes should be available to P/FM on the hospital units
**Visitors' restrictions**	• FM should replace their visits by virtual visits/phone calls<br>• FM should not limit their visit at the hospital<br>• FM have the role and responsibility to limit their visit, but should not stop visiting<br>• Visits should be prohibited and replaced by virtual visits<br>• Only a limited number of FM should be allowed to visit<br>• All FM should be allowed to visit without exception<br>• HP should encourage FM to do a virtual visit/phone call<br>• HP should ask FM questions prior visiting<br>• FM should sign a document that commits them to respect IPV prior visiting

[1]Patients (P); [2]Family members (PM); [3]Infection prevention measures (IPM); [4]Healthcare professionals (HP); [5]Hand hygiene measure (HHM); [6]Patient partners (PP)

Clavel NC, *et al. BMJ Open* 2022; 12:e056172. doi: 10.1136/bmjopen-2021-056172
