# Supplementary material for: Patient and family engagement in infection prevention in the context of the COVID-19 pandemic: defining a consensus framework using the Q methodology – NOSO-COVID study protocol
Source: BMJ Open. 2022 Jul 22;12(7):e056172. doi: 10.1136/bmjopen-2021-056172 (PMC9315236; doi:10.1136/bmjopen-2021-056172)
Supplement: Author's manuscript [file bmjopen-2021-056172.draft_revisions.pdf]

BMJ Open is committed to open peer review. As part of this commitment we make the peer review history of every article we publish publicly available.

When an article is published we post the peer reviewers' comments and the authors' responses online. We also post the versions of the paper that were used during peer review. These are the versions that the peer review comments apply to.

The versions of the paper that follow are the versions that were submitted during the peer review process. They are not the versions of record or the final published versions. They should not be cited or distributed as the published version of this manuscript.

BMJ Open is an open access journal and the full, final, typeset and author-corrected version of record of the manuscript is available on our site with no access controls, subscription charges or pay-per-view fees (http://bmjopen.bmj.com).

If you have any questions on BMJ Open's open peer review process please email info.bmjopen@bmj.com

BMJ Open

Patient and Family Engagement in Infection Prevention in the Context of the COVID-19 pandemic: Defining a consensus framework using Q-Methodology - NOSO-COVID study protocol

Journal:	*BMJ Open*
Manuscript ID	bmjopen-2021-056172
Article Type:	Protocol
Date Submitted by the Author:	05-Aug-2021

Complete List of Authors:	Clavel, Nathalie; McGill University Faculty of Medicine, Ingram School of Nursing<br>Lavoie-Tremblay, Mélanie ; McGill University Faculty of Medicine, Ingram School of Nursing<br>Biron, Alain; McGill University Health Centre<br>Briand, Anaick ; McGill University Health Centre<br>Paquette, Jesseca; McGill University Faculty of Medicine, Ingram School of Nursing<br>Bernard, Laurence ; University of Montreal, Faculty of Nursing<br>Fancott, Carol; Healthcare Excellence Canada, Patient Engagement & Partnerships<br>Pomey, Marie-Pascale; Universite de Montreal,<br>Dumez , Vincent; Centre of Excellence on Partnership with patients and the public

Keywords:	Health & safety < HEALTH SERVICES ADMINISTRATION & MANAGEMENT, Quality in health care < HEALTH SERVICES ADMINISTRATION & MANAGEMENT, Risk management < HEALTH SERVICES ADMINISTRATION & MANAGEMENT

SCHOLARONE™ Manuscripts

Patient and family engagement in infection prevention in the context of the COVID-19 pandemic: Defining a consensus framework using the Q methodology - NOSO-COVID study protocol

Running title: NOSO-COVID study - Patients & families engaged against nosocomial infections in the context of COVID-19

Nathalie Clavel[1], Mélanie Lavoie-Tremblay[1], Alain Biron[1,2], Anaïck Briand[2], Jesseca Paquette[1], Laurence Bernard[3], Carol Fancott[4], Marie-Pascale Pomey[5,6,7], Vincent Dumez[7,8]

Corresponding author: Nathalie Clavel, Postdoctoral Fellow, Ingram School of Nursing, McGill University; nathalie.clavel@mail.mcgill.ca, +1514 999 0453

1: Ingram School of Nursing, McGill University, Montreal, Canada

2: Nursing Department, McGill University Health Center, Montreal, Canada

3: Faculté des Sciences Infirmières, Université de Montréal, Montreal, Canada

4: Patient Engagement & Partnerships, Healthcare Excellence Canada

5: Department of management, evaluation and policy, School of Public Health, Université de Montréal, Montreal, Canada

6: Centre de recherche du Centre Hospitalier Universitaire de Montréal, Canada

7: Centre of Excellence on Partnership with patients and the public, Montreal, Canada

8: Faculty of Medicine, Université de Montréal, Montreal, Canada

Keywords: patient and family engagement, patient safety, healthcare-associated infections, prevention and control, Q methodology

Word count: 4097

For peer review only

ABSTRACT (255 words)

Introduction

Healthcare-associated infections are an important patient safety concern, especially in the context of the COVID-19 pandemic. Infection prevention and control implemented in healthcare settings are largely focused on the practices of healthcare professionals. Patient and family engagement is also recognized as an important patient safety strategy. The extent to which patients and families can be engaged, their specific roles, and the strategies that support their engagement in infection prevention remain unclear. The overarching objective of the proposed study is to explore how patients and families can effectively be engaged in infection prevention by developing a consensus framework with key stakeholders.

Design and Methods

The proposed study is based on a cross-sectional exploratory study at one of the largest university hospitals in North America (Montreal, Canada). The targeted population is all healthcare professionals, managers, and other non-clinical staff members who work on clinical units, and the in-patients and their families. The study is based on Q methodology that takes advantage of both quantitative and qualitative methods to identify the consensus among the various stakeholders. This exploratory Q research approach will provide a structured way to elicit the stakeholders' perspectives on patient and family engagement in infection prevention.

Ethics and Dissemination

The Research Ethics Board of the participating university hospital approved this study. The research team plans to disseminate the findings through different channels of communication targeting healthcare professionals, managers in healthcare settings, and patients and family caregivers. The findings will also be disseminated through peer-reviewed journals in healthcare management and in quality and safety improvement.

For peer review only - http://bmjopen.bmj.com/site/about/guidelines.xhtml

Strengths and Limitations of the Study

- The study is based on Q methodology, which is relevant for exploring different perspectives and for identifying the consensus among various stakeholders for a set of strategies and actions to be implemented in healthcare settings.
- The proposed Q study takes advantage of quantitative and qualitative research methods to explore and understand in a structured and in-depth way the perspectives of different key stakeholders on patient and family engagement in infection prevention.
- The study will be conducted with key stakeholders who play a role in infection prevention and control (patients, family caregivers, healthcare professionals, managers, and other non-clinical staff members).

  The study will unfold within a single healthcare organization, which may limit the generalizability of the findings. However, these findings could support other organizations to create their own frameworks for engagement practices in infection prevention and control.
- This study will be conducted in different clinical units to compare the different contexts of care.

For peer review only - http://bmjopen.bmj.com/site/about/guidelines.xhtml

Introduction

Healthcare-associated infections (HAIs) are the most frequent complications for patients receiving healthcare. The threat requires increased vigilance in healthcare settings. Internationally, 5% to 15% of patients admitted to hospitals acquire an infection (1). In Canada, HAIs remain particularly persistent with more than 200,000 Canadians being infected every year while receiving healthcare (2). HAIs are infections that are acquired in patients in settings where care is delivered, including healthcare settings (e.g., hospitals or long-term facilities) (3). Examples of bacterial HAIs that are commonly found in healthcare settings include methicillin-resistant Staphylococcus aureus (MRSA) infections, vancomycin-resistant enterococci (VRE) infections, and Clostridium difficile (C. difficile) infections (1, 4). These infections have major health-related and financial consequences for health systems, including higher mortality and morbidity rates and increased lengths of stay at hospitals (5-7).

In the current context of the spread of SARS-CoV-2 in the community, with asymptomatic people spreading the virus, and a proportion of infected people being hospitalized, the incidence of HAIs is expected to increase in the coming months and years. One rapid review and meta-analysis showed that 44% of the confirmed cases of COVID-19 were acquired within healthcare settings (8). Another paper highlighted the risk for inpatients with COVID-19 to have HAIs as secondary infections. Moreover, in adult inpatients diagnosed with COVID-19 in Wuhan, China, half of the non-survivors experienced a secondary infection (9). SARS-CoV-2 is highly contagious and can be rapidly spread in healthcare settings if no specific prevention and control measures are implemented and sustained over time. Thus, the involvement and engagement of patients and their families is needed to prevent spread.

However, to date, infection, prevention, and control (IPAC) strategies implemented in healthcare settings have largely focused on the practices of healthcare professionals (HCPs), (e.g., nurses, physicians, allied health professionals) and other staff members (e.g., housekeepers, transport attendants, unit managers), without defining how patients and their relatives could be involved to reduce the infections. Patient and family engagement is

widely recognized as a promising strategy for improving the quality of healthcare and patient safety (10-13). In recent years, several studies have examined patient engagement in IPAC (14-17). In the literature, a consensus has been expressed that patients, family caregivers, and health professionals should play joint roles in preventing HAIs. Nevertheless, the extent to which patients and families can effectively be engaged and their specific roles and responsibilities in IPAC remain unclear (18, 19). The evidence suggests that patients may feel anxious or uncomfortable about asking about or getting involved in their safety (20). Studies have also reported that health professionals often acknowledge patients as being vulnerable though not necessarily co-responsible for preventing infection transmission (18). Finally, few studies have identified existing strategies to engage patients and family caregivers in IPAC (18), though developing targeted strategies to enable patient and family engagement might contribute to enhance IPAC activities.

In line with the recommendations of the World Health Organization (21) and the action plan of the Quebec Ministry of Health and Social Services (22), a Montreal university hospital implemented an interdisciplinary organizational auditing program that aims to reduce HAIs. This nurse-led program, implemented in 2016 in several clinical units focuses on increasing the compliance of: a) hand hygiene, b) appropriate glove use, c) appropriate personal protective equipment use, and d) proper cleaning and disinfection of environment and equipment. To achieve these objectives, four main strategies were implemented: 1) staff training on IPAC, IPAC tools, and process improvements, 2) audits of hand hygiene, 3) glove use and disinfection of equipment, and 4) communication reinforcement on IPAC among staff using huddles (23). Despite improvements in nosocomial infection rates and hand hygiene compliance among staff from 37% to 67% (23), audits on clinical units showed that hand hygiene rates among patients and visitors remained low (under 50%). The strategies that were implemented specifically targeted the practices of healthcare professionals (23), without defining how patients and family caregivers could be involved to reduce the infections.

During the pandemic, healthcare organizations have implemented new measures that target patients and family caregivers visiting patients. Some of these measures have increased

For peer review only - http://bmjopen.bmj.com/site/about/guidelines.xhtml

patient and family responsibilities in infection prevention (e.g., routine hand hygiene, systematic wearing of masks), while other measures have limited the family caregivers' essential roles as partners (e.g., prohibition or limitation of non-essential visits) (24, 25). These restrictive policies were enacted quickly as the pandemic emerged, without input from all stakeholders (including patients, family caregivers, clinical and non-clinical staff members) who play a major role in IPAC. Thus, creating a consensus framework will help to define the patients' and family caregivers' roles and responsibilities in IPAC during a pandemic and identify the strategies for promoting and facilitating their engagement in this important area of patient safety.

Aims and Objectives

To address the gap, the overarching objective of the study is to explore how patients and families can effectively be engaged in IPAC in the context of the COVID-19 pandemic. Specifically, the study is based on the following objectives:

Objective 1: to measure and understand patients' and families' preferences regarding their engagement in IPAC.

Objective 2: to measure and understand the preferences of clinical and non-clinical staff on patient and family engagement in IPAC.

Objective 3: to identify a consensus framework for the roles and responsibilities of patients and families in IPAC and strategies to facilitate their engagement.

Methods and Analysis

Project Design and Research Approach

The proposed study uses a cross-sectional exploratory approach. The targeted population is comprised of all clinical and non-clinical staff members who work in clinical units of the participating healthcare organization that have implemented the interdisciplinary organizational IPAC program, and patients hospitalized in these units and their families or other caregivers. The study is based on the Q methodology (26), an exploratory research approach that provides a structured way to elicit the stakeholders' perspectives on the issue by using both qualitative and quantitative methods. It offers an innovative alternative to traditional methods for statistically analyzing a variety of perspectives that can lead to the

For peer review only - http://bmjopen.bmj.com/site/about/guidelines.xhtml

development of a consensus on a set of actions, interventions, or policies for implementation (26, 27).

Project Setting and Stages

The research project will take place at an academic health network that is one of the largest in North America. The healthcare organization provides tertiary and quaternary care to the population of Montreal and Quebec. The project will be divided into three stages that have been identified as feasible by key stakeholders at the participating healthcare organization.

The Q study is divided into three successive stages (Figure 1). The different sub-sections in the methods section will be presented for each stage successively.

Insert Figure 1

Conceptual Framework

The study relies on a theoretical framework adapted from Pomey et al. (28) on patient and citizen engagement in healthcare systems and on the work of Arnstein (29) on citizen participation in public policy (Figure 2). The framework describes the spectrum of patient and family engagement in infection prevention from paternalism to partnership (co-responsibility). The higher level of engagement involves supporting a shared responsibility among patients, families, and clinical and non-clinical staff members towards infection prevention. At the extreme opposite of the spectrum, mechanisms can be implemented to ensure that patients and families comply with infection prevention measures or are informed about infection prevention practices in hospital settings.

Insert Figure 2

For peer review only - http://bmjopen.bmj.com/site/about/guidelines.xhtml

Stage 1– Elaboration of a set of statements with key experts (fall 2020 - winter 2021)

Objective. The preparatory phase of the study will consist of elaborating on a sample of statements (Q set) on patient and family engagement in IPAC that will be used in the pilot Q study.

Setting and Participants

The participants will be comprised of key stakeholders involved in the interdisciplinary organizational program focusing on IPAC and managers and coordinators responsible for patient and family engagement at the participating healthcare organization. Stakeholders involved in IPAC will be recruited through the steering committee of the interdisciplinary organizational program responsible for reflecting, assessing, and orientating appropriate practices in IPAC. The program has been ongoing since 2016 and more than 30 units have implemented the strategies targeting improvement in IPAC practices within the organization. The steering committee brings together healthcare professionals (including clinical managers) and members from different departments who have a key role regarding IPAC (i.e., nursing, medical affairs, infection control, housekeeping, and quality). In addition, managers and coordinators of patient and family engagement will be recruited through the quality department that is responsible for implementing patient engagement practices within the organization. We seek to recruit a total of 10 participants and plan to organize a focus group with each group of stakeholders (five experts per group: members of the steering committee on IPAC and managers/coordinators of patient engagement).

Data Collection and Analysis

Statements on patient and family engagement in IPAC will be drawn from two sources. First, a literature review of scientific papers and gray literature will be conducted to highlight key findings in the area of patient engagement in IPAC, including the specific context of the COVID-19 pandemic. Second, the qualitative data derived from two focus groups conducted with key experts in IPAC and patient and family engagement will be used. Data from these two sources will be integrated to draw a list of approximately 40 to

For peer review only - http://bmjopen.bmj.com/site/about/guidelines.xhtml

60 statements that represent various perspectives on patient and family engagement in IPAC.

Stage 2 – Pilot Q study in one clinical unit (spring and summer 2021)

Objective. This stage will help to measure and understand the preferences of patients, family caregivers, and staff members on patient and family engagement in IPAC (Objectives 1 and 2).

This stage will help to identify different groups of perspectives regarding the roles of patients and their families in IPAC and the strategies/actions that can be implemented to enhance the preferred roles.

Setting and Participants

This stage will be conducted on one of the clinical units (adult surgery) that has implemented the interdisciplinary program focusing on IPAC. This unit has been chosen for two reasons: (1) the unit has shown low hand hygiene compliance rates from patients and visitors before the pandemic (37% in 2019) and (2) patients hospitalized in this unit are immunosuppressed and then need greater protection from infections. The targeted total sample will be 30 participants: 15 members of the clinical unit (health professionals, managers, and other non-clinical staff members) and 15 patients or family caregivers. The recruitment of patients, family caregivers, and staff members will be carried out by the nurse manager and the assistant nurse manager of the unit.

Inclusion criteria. All staff currently working in the clinical unit (HCPs, managers, and other staff members) and who are willing to participate will be included in the study. Patients who have been hospitalized or relatives whose loved ones have been hospitalized on these units will be considered for inclusion if they meet the following conditions: (1) 18 years or older; (2) have been hospitalized on the unit for two months prior to the time of data collection; (3) speak and read French or English; (4) have no serious cognitive or

For peer review only - http://bmjopen.bmj.com/site/about/guidelines.xhtml

speech impairments, or any other conditions that would limit their ability to provide informed consent, and (5) have access to the Internet.

Data Collection

In the pilot Q study, data collection will be based on two methods used in Q methodology (Q surveys and interviews), which will be done remotely because of the COVID-19 pandemic.

All participants will be invited to complete the Q online survey developed in Stage 1. The Q survey will consist of socio-demographic information questions and 40 to 60 statements that participants will have to sort and rank according to a two-step sorting and ranking process specific to the Q methodology. This first step will consist of sorting each statement into three categories (agree, neutral, or disagree), and then a second step will ask participants to prioritize statements by ranking them in five categories (-2 completely disagree, -1 disagree, 0 neutral, +1 agree, +2 completely agree). A forced-choice distribution of statements will allow the participants to add a limited and fixed number of statements for each category (4 items in +2/-2, 8 items in +1/-1, 16 items in 0). The Q survey will be hosted on a specific platform (QsorTouch) that has been developed for Q methodologist researchers to easily and securely administrate Q online surveys.

Post-sorting/ranking interviews will be conducted with participants after they have completed the survey and after the researcher has reviewed each participant's Q sort (ranking of statements). As part of the interviews, participants will be asked to explain the rationale of ranking statements in the most extreme scales (i.e., - 2 or +2) and some of the statements ranked in the other scales (0 and -1/+1). Phone interviews will last approximately 30 min and will be audio-recorded.

Data Analysis

Quantitative data from Q surveys. At stage 2, data analysis will consist of comparing and grouping by similarity all of the Q sorts that are collected. The number of Q sorts will be equal to the number of participants (n=30). A by-person factor analysis will be undertaken

For peer review only - http://bmjopen.bmj.com/site/about/guidelines.xhtml

to measure the intercorrelation of all of the gathered Q sorts to identify a group of persons who ranked the order of statements in a very similar fashion (Q factors) (30). A factor in Q methodology represents a group of persons who share a similar perspective or viewpoint about patient and family engagement in IPAC. Grouping, comparing, and summarizing the Q sorts will be performed using data-reduction techniques, including principal component analysis (PCA). The analysis will be done using PQMethods software, which is dedicated to analyzing Q data. PCA consists of reducing the data to a few factors. The reduction in PCA will be done according to two steps: extraction and rotation. Extraction consists of summarizing all of the participants' responses into a few representative responses – the Q perspectives. Rotation is used to have a clearer structure of the results, including an understanding of the percentage of variability explained by the Q perspective (30). In this study, an example of a Q perspective could be participants in favor of controlling patients and family members' compliance with IPAC practices. Factor loadings will be calculated to explain the relationship between each participant and factor (Q perspective). Z scores will also be examined to "give more precision about how strongly engaged each perspective is with each item" (26).

Qualitative data from interviews. Transcriptions from the interviews will be analyzed using QDA Miner. A thematic analysis will be performed based on our conceptual framework (Figure 2) and following Miles and Huberman methods for qualitative data analysis (Miles & Huberman, 2014).

Integration of quantitative and qualitative data. The interpretation of the results will be based on both quantitative data and qualitative data. Each factor will be described in detail and given a meaningful label to indicate what similar perspectives are about; for example: "controlling patients and family members' IPAC practices" or "supporting shared responsibility among patients, family members, and staff members for IPAC." Each perspective will reveal and describe the preferences of participants regarding patient and family engagement in IPAC. The in-depth qualitative data will allow for an understanding of the rationale of each perspective.

For peer review only - http://bmjopen.bmj.com/site/about/guidelines.xhtml

Stage 3 – Validation in four other clinical units (fall 2021- winter 2022)

Objective. This stage of the study is designed to validate the pilot Q findings from a larger sample of clinical units. Since the pilot Q study is based on a small sample, it will not be possible to generalize the findings to a larger population (e.g., other clinical units). This can be done by combining a Q study methodology with an R methodology (traditional survey method) (31).

Setting and Participants

The four participating units at the MUHC will be randomly selected. All staff members (clinical and non-clinical) and patients and family caregivers will be invited to participate in the study. All potential participants will be given a package containing a letter of introduction describing the study, a demographic survey, and a link to the survey that will be administrated online.

In Stage 3, the targeted survey sample of participants is 200 participants, equally distributed among the four clinical units that will be randomly selected: 70 patients, 30 family caregivers, 70 HCPs (including managers), 30 non-clinical staff members (housekeepers, transport attendants, etc.). The inclusion criteria for the patients and family caregivers will be the same as for Stage 2.

Data Collection

Creation and administration of a traditional survey. To create a traditional survey, several highly and weakly ranked distinctive statements from each Q perspective measured at Stage 2 will be included and presented as Likert items (a 5-point Likert scale). This technique is the most commonly used for combining Q methodology and survey methods (31, 32). The participants in each clinical unit will be invited to complete the online survey.

Data Analysis

The survey data will be entered in MS-Excel and then exported and analyzed using SPSS software.

For peer review only - http://bmjopen.bmj.com/site/about/guidelines.xhtml

Any returned surveys that are missing one or more responses will be excluded from the analysis. For each sample (each clinical unit), we will perform descriptive statistics, calculating mean scores, standard deviations, and the percentage of agreement for each item and the overall scale. High score items shared in the four samples will be identified to develop a consensus framework on the roles of patients and families and strategies that enhance their engagement in IPAC. In addition, item scores will be compared across the samples (clinical units) for type of respondents (patients, relatives, HCPs, managers, and other non-clinical staff members), and across different demographic characteristics (age, gender, and level of education). The comparisons will help to identify shared preferences across units, in terms of patient and family roles and strategies to enhance their engagement in IPAC. The comparisons will also help to show differences between the participants and clinical units in terms of preferences for engaging patients and families in IPAC.

Patient and Public Involvement

Patient advisors will be involved in the pilot testing of the online surveys and in the different stages of the study to have their inputs and feedback on the different tools used for data collection and the preliminary results (Stages 1, 2 and 3). One of the collaborators for this study is a patient expert who is a leading member of a reference Canadian center in patient and family engagement.

Discussion

To date, little is known about how patients and families could be effectively engaged in IPAC, an important area of patient safety (18, 33, 34). Patient and family engagement in infection prevention remains unclear and is subject to barriers that have been studied previously (18, 35, 36). Moreover, the COVID-19 pandemic has raised important trade-offs between patient safety/IPAC practices on one hand and patient and family engagement as essential partners in the other hand (37-39). Developing targeted strategies to overcome the barriers associated with patient engagement is important for patient engagement to become a core component of IPAC programs. Engaging patients in IPAC can help reduce the burden of HAIs in clinical units and healthcare settings. Because of the complex reactions and barriers regarding patient engagement in patient safety, this study will help

For peer review only - http://bmjopen.bmj.com/site/about/guidelines.xhtml

define the "optimal" roles of patients in IPAC and targeted strategies that are acceptable for stakeholders, including patients, family caregivers, HCPs, managers, and other non-clinical staff members.

The proposed Q study is based on an innovative methodological approach (the Q methodology) that takes advantage of qualitative and quantitative methods to explore the subjectivity of perceptions in a statistical way. Using this methodology is particularly relevant to understand in a structured and clear way the various perspectives on patient and family engagement in IPAC. To date, current studies on patient engagement in IPAC have focused on qualitative methods that provide few insights on consensus strategies among the many actors in the area of patient engagement in patient safety and IPAC.

Potential Implications for Healthcare Settings, Patients, and Families

This study can contribute to improving IPAC programs that are currently focused on HCP best practices in IPAC, including hand hygiene. During this time of pandemic, the importance of engaging all stakeholders in IPAC processes is paramount. This research will help develop additional and targeted strategies to better engage patients and their families in patient safety, in the important area of infection prevention. Based on several clinical units, the Q study will also help to elaborate tailor-made strategies of patient engagement in IPAC in different clinical contexts.

Ethics and Dissemination

Ethical and Data Protection Considerations

Written consent will be obtained from all participants before each interview. Ethical approval for the current study was obtained from the X Research Ethics Board (Patient and Family Engagement / 2020-6404). Electronic data: i.e., information and consent forms, quantitative data from surveys, digital recordings, and interview transcriptions will be password protected and only accessible by members of the research team. Transcription data will be anonymized and audio-recorded interviews destroyed after they are transcribed.

For peer review only - http://bmjopen.bmj.com/site/about/guidelines.xhtml

Outputs and Dissemination

This study has been awarded funding by the Social Sciences and Humanities Research Council (SSHRC) as part of the Partnership Engage Grants (PEG) for which we developed a partnership with Healthcare Excellence Canada and a collaboration with the Center of Excellence on Partnership with Patients and the Public (CEPPP). The pandemic has challenged many of the principles of patient partnership in care and the inclusion of family caregivers as essential partners in care, especially with the restrictive visiting policies. In Canada and abroad, the pandemic reminds us of the essential presence of family caregivers to continue to provide moral and physical support particularly to vulnerable patients such as older adults and children. In this context, Healthcare Excellence Canada and CEPPP are particularly committed to finding effective ways to better involve patients in IPAC and to reintegrate the family presence in healthcare settings. Such a framework will be especially useful for Healthcare Excellence Canada in their 'Better Together' program that focuses on reintegrating family caregivers as essential partners in care. The framework could also be used as a tool designed for health professionals and managers, and patients and family caregivers to help guide and support inclusive engagement initiatives in IPAC in the context of a pandemic and beyond.

As part of our partnership and collaboration, various knowledge mobilization activities, including knowledge synthesis, dissemination, and exchange and co-creation with knowledge users and partners will be co-developed. Dissemination activities and approaches will leverage the networks and existing structures of our partners, including Healthcare Excellence Canada and CEPPP, to broadly share the co-created framework to health decision-makers, healthcare professionals, patients, and family caregivers. The research findings will be presented and submitted to international quality and safety management and improvement conferences and journals.

Conclusion

This study will increase our understanding and knowledge on how patients and families could be engaged in infection prevention in the context of a pandemic. The development of a consensus framework on patient and family engagement in IPAC will help to define

For peer review only - http://bmjopen.bmj.com/site/about/guidelines.xhtml

the roles of patients and families and strategies that support their engagement in this important area of patient safety.

Authors' Contributions

The study concept and design were conceived by NC with substantial feedback from MLT, ABI, and ABR. NC and JP will conduct the data collection and analysis. NC prepared the first draft of the manuscript, and all authors provided edits and critiqued the manuscript for intellectual content. All authors read and approved the final manuscript.

Acknowledgements

We thank the Social Sciences and Humanities Research Council for providing research funding for this study. The authors also thank Glen Wheeler for his contribution to the editing of this manuscript.

Funding Statement

This study has received funding from the Social and Sciences Humanities Research Council (Government of Canada) through the Partnership Engage Grant – a special initiative for COVID-19 (SSHRC 1008-2020-1062). This study is also supported by a postdoctoral scholarship to X from the *Fonds de Recherche du Québec- Santé*. Finally, research funds were provided by the McGill University Health Center and the McGill Nursing Collaborative for Education and Innovation in Patient- and Family-Centred Care (Newton Foundation/McGill Faculty of Medicine) (N/A).

Data-Sharing Statement

The final dataset will be available to study investigators, and to the Research Ethic Boards at the participating site.

Competing Interests' Statement

None declared.

For peer review only - http://bmjopen.bmj.com/site/about/guidelines.xhtml

Figures

Figure 1: Study stages

Figure 2: Conceptual framework

For peer review only

For peer review only - http://bmjopen.bmj.com/site/about/guidelines.xhtml

References

1. Haque M, Sartelli M, McKimm J, Abu Bakar M. Health care-associated infections - an overview. Infect Drug Resist. 2018;11:2321-33.
2. Health Canada and the Public Health Agency of Canada. Evaluation of Healthcare-Associated Infection Activities at the Public Health Agency of Canada 2012-13 to 2016-17. Ottawa Health Canada; 2018.
3. World Health Organization. Report on the Burden of Endemic Health Care-Associated Infection Worldwide. Geneva World Health Organization 2011.
4. World Health Organization. Drug resistance Geneva World Health Organization; 2021 [Available from: https://www.who.int/drugresistance/AMR_Importance/en/.
5. De Angelis G, Murthy A, Beyersmann J, Harbarth S. Estimating the impact of healthcare-associated infections on length of stay and costs. Clin Microbiol Infect. 2010;16(12):1729-35.
6. Calfee DP. Crisis in Hospital-Acquired, Healthcare-Associated Infections. Annu Rev Med. 2012;63(1):359-71.
7. Tchouaket Nguemeleu E, Beogo I, Sia D, Kilpatrick K, Séguin C, Baillot A, et al. Economic analysis of healthcare-associated infection prevention and control interventions in medical and surgical units: Systematic review using a discounting approach. J Hosp Infect. 2020.
8. Zhou Q, Gao Y, Wang X, Liu R, Du P, Wang X, et al. Nosocomial infections among patients with COVID-19, SARS and MERS: a rapid review and meta-analysis. Ann Transl Med. 2020;8(10):629-.
9. Zhou F, Yu T, Du R, Fan G, Liu Y, Liu Z, et al. Clinical course and risk factors for mortality of adult inpatients with COVID-19 in Wuhan, China: a retrospective cohort study. The Lancet. 2020;395(10229):1054-62.
10. Berger Z, Flickinger TE, Pfoh E, Martinez KA, Dy SM. Promoting engagement by patients and families to reduce adverse events in acute care settings: a systematic review. BMJ Qual Saf. 2014;23(7):548-55.
11. Canadian Patient Safety Institute. Engaging Patients in Patient Safety - a Canadian Guide. Ottawa: Canadian Patient Safety Institute, ; 2017.
12. Kushner C, Davis D. Improving safety: engaging with patients and families makes a difference! Healthc Q. 2014;17 Spec No:41-4.
13. Pomey M-P, Clavel N, Aho-Glele U, Ferré N, Fernandez-McAuley P. How patients view their contribution as partners in the enhancement of patient safety in clinical care Patient Experience Journal 2018;5(1):35-49.
14. Allegranzi B, Conway L, Larson E, Pittet D. Status of the implementation of the World Health Organization multimodal hand hygiene strategy in United States of America health care facilities. Am J Infect Control. 2014;42(3):224-30.
15. Seale H, Chughtai AA, Kaur R, Phillipson L, Novytska Y, Travaglia J. Empowering patients in the hospital as a new approach to reducing the burden of health care-associated infections: The attitudes of hospital health care workers. Am J Infect Control. 2016;44(3):263-8.
16. Pan SC, Tien KL, Hung IC, Lin YJ, Yang YL, Yang MC, et al. Patient empowerment in a hand hygiene program: differing points of view between patients/family members and health care workers in Asian culture. Am J Infect Control. 2013;41(11):979-83.
17. Butenko S, Lockwood C, McArthur A. Patient experiences of partnering with healthcare professionals for hand hygiene compliance: a systematic review. JBI Database System Rev Implement Rep. 2017;15(6):1645-70.

For peer review only - http://bmjopen.bmj.com/site/about/guidelines.xhtml

18. Fernandes Agreli H, Murphy M, Creedon S, Ni Bhuachalla C, O'Brien D, Gould D, et al. Patient involvement in the implementation of infection prevention and control guidelines and associated interventions: a scoping review. BMJ open. 2019;9(3):e025824.
19. Bellissimo-Rodrigues F, Pires D, Zingg W, Pittet D. Role of parents in the promotion of hand hygiene in the paediatric setting: a systematic literature review. J Hosp Infect. 2016;93(2):159-63.
20. Alzyood M, Jackson D, Brooke J, Aveyard H. An integrative review exploring the perceptions of patients and healthcare professionals towards patient involvement in promoting hand hygiene compliance in the hospital setting. J Clin Nurs. 2018;27(7-8):1329-45.
21. World Health Organization. Guidelines on Core Components of Infection Prevention and Control Programmes at the National and Acute Health Care Facility Level. Geneva 2016.
22. Ministère de la santé et des services sociaux. Prévention et contrôle des infections nosocomiales. Plan d'action 2010-2015. Québec Ministère de la santé et des services sociaux,; 2011.
23. Bernard L, Biron A, Briand A, Taha S, Lavoie-Tremblay M. Evaluation of a quality improvement program to prevent healthcare acquired infections in an acute care hospital. Journal of Nursing Education and Practice 2021;11(5):24-31.
24. World Health Organization. Infection prevention and control during health care when COVID-19 is suspected Geneva World Health Organization; 2020.
25. Institut National de la Santé Publique du Québec. Mesures de prévention et contrôle des infections pour les milieux de soins aigus : recommandations intérimaires. Montreal, Quebec: Institut National de la Santé Publique du Québec; 2020.
26. Zabala A, Sandbrook C, Mukherjee N. When and how to use Q methodology to understand perspectives in conservation research. Conserv Biol. 2018;32(5):1185-94.
27. Alderson S, Foy R, Bryant L, Ahmed S, House A. Using Q-methodology to guide the implementation of new healthcare policies. BMJ Qual Saf. 2018;27(9):737-42.
28. Pomey M, Flora L, Karazivan P, Dumez V, Lebel P, Vanier M-C, et al. Le "Montreal Model": enjeux du partenariat relationnel entre patients et professionnels de la santé Sante Publique (Bucur). 2015;HS(S1):41-50.
29. Arnstein SR. A Ladder Of Citizen Participation. J Am Inst Plann. 1969;35(4):216-24.
30. Watts S, Stenner P. Doing Q Methodological Research SAGE, editor. London SAGE; 2012. 238p p.
31. Danielson S. Q Method and Surveys: Three Ways to Combine Q and R. Field Methods. 2009;21(3):219-37.
32. Brown SR. Political Subjectivity: Applications of Q Methodology in Political Science . New Haven Yale University Press; 1980.
33. Bishop AC, Macdonald M. Patient Involvement in Patient Safety: A Qualitative Study of Nursing Staff and Patient Perceptions. Journal of patient safety. 2017;13(2):82-7.
34. Abbasgholizadeh Rahimi S, Zomahoun HTV, Légaré F. Patient Engagement and its Evaluation Tools – Current Challenges and Future Directions; Comment on "Metrics and Evaluation Tools for Patient Engagement in Healthcare Organization- and System-Level Decision-Making: A Systematic Review". IJHPM. 2019;8(6):378-80.
35. Landers T, Abusalem S, Coty M-B, Bingham J. Patient-centered hand hygiene: The next step in infection prevention. Am J Infect Control. 2012;40(4, Supplement):S11-S7.
36. McGuckin M, Govednik J. Patient empowerment and hand hygiene, 1997-2012. J Hosp Infect. 2013;84(3):191-9.

For peer review only - http://bmjopen.bmj.com/site/about/guidelines.xhtml

37. Canadian Foundation for Healthcare Improvement. Much More than A Visitor: An Executive Summary of Policies in Canadian Acute Care Hospital. Ottawa Canadian Foundation for Healthcare Improvement 2020.
38. Canada Foundation for Healthcare Improvement, Canadian Patient Safety Institute. EVIDENCE BRIEF: Caregivers as Essential Care Partners. Ottawa Canada Foundation for Healthcare Improvement, Canadian Patient Safety Institute; 2020.
39. Canadian Foundation for Healthcare Improvement. BETTER TOGETHER: ReIntegration of Family Caregivers as Essential Partners in Care in a Time of COVID-19 Canada Foundation for Healthcare Improvement; 2020.

For peer review only

For peer review only - http://bmjopen.bmj.com/site/about/guidelines.xhtml

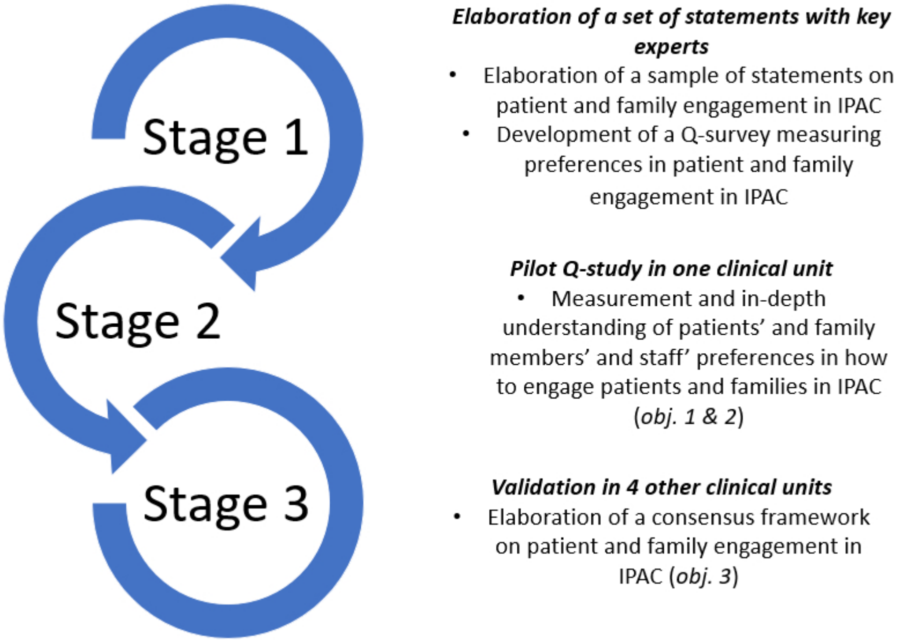

Figure 1_ study stages

385x293mm (57 x 57 DPI)

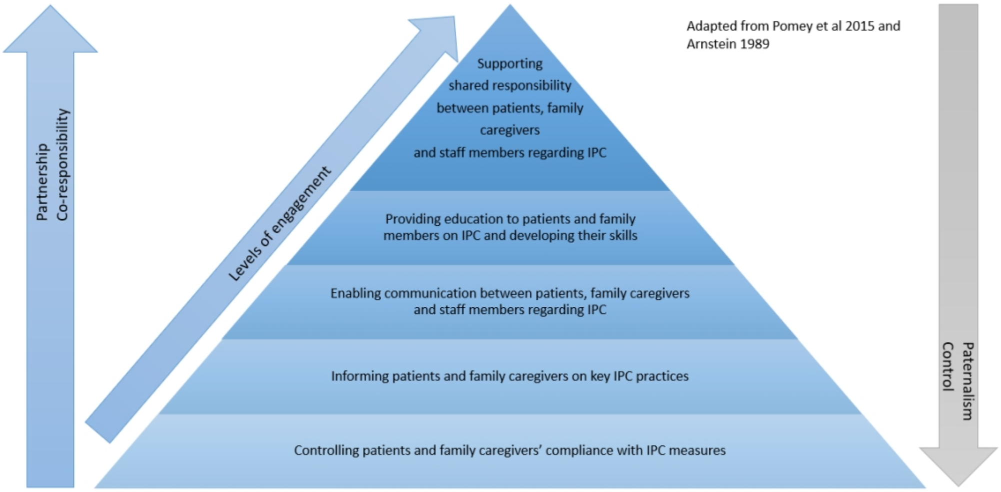

Figure 2_conceptual framework

569x301mm (57 x 57 DPI)

BMJ Open

Patient and family engagement in infection prevention in the context of the COVID-19 pandemic: Defining a consensus framework using the Q methodology - NOSO-COVID study protocol

Journal:	*BMJ Open*
Manuscript ID	bmjopen-2021-056172.R1
Article Type:	Protocol
Date Submitted by the Author:	14-Apr-2022
Complete List of Authors:	Clavel, Nathalie; McGill University Faculty of Medicine, Ingram School of Nursing<br>Lavoie-Tremblay, Mélanie ; McGill University Faculty of Medicine, Ingram School of Nursing<br>Biron, Alain; McGill University Health Centre<br>Briand, Anaick ; McGill University Health Centre<br>Paquette, Jesseca; McGill University Faculty of Medicine, Ingram School of Nursing<br>Bernard, Laurence ; University of Montreal, Faculty of Nursing<br>Fancott, Carol; Healthcare Excellence Canada, Patient Engagement & Partnerships<br>Pomey, Marie-Pascale; Universite de Montreal,<br>Dumez , Vincent; Centre of Excellence on Partnership with patients and the public
**Primary Subject Heading**:	Health services research
Secondary Subject Heading:	Infectious diseases
Keywords:	Health & safety < HEALTH SERVICES ADMINISTRATION & MANAGEMENT, Quality in health care < HEALTH SERVICES ADMINISTRATION & MANAGEMENT, Risk management < HEALTH SERVICES ADMINISTRATION & MANAGEMENT

SCHOLARONE™
Manuscripts

Patient and family engagement in infection prevention in the context of the COVID-19 pandemic: Defining a consensus framework using the Q methodology - NOSO-COVID study protocol

Nathalie Clavel[1], Mélanie Lavoie-Tremblay[1], Alain Biron[1,2], Anaïck Briand[2], Jesseca Paquette[1], Laurence Bernard[3], Carol Fancott[4], Marie-Pascale Pomey[5,6,7], Vincent Dumez[7,8]

Corresponding author: Nathalie Clavel, Postdoctoral Fellow, Ingram School of Nursing, McGill University; nathalie.clavel@mail.mcgill.ca

1: Ingram School of Nursing, McGill University, Montreal, Canada

2: Nursing Department, McGill University Health Center, Montreal, Canada

3: Faculté des Sciences Infirmières, Université de Montréal, Montreal, Canada

4: Patient Engagement & Partnerships, Healthcare Excellence Canada

5: Department of management, evaluation and policy, School of Public Health, Université de Montréal, Montreal, Canada

6: Centre de recherche du Centre Hospitalier Universitaire de Montréal, Canada

7: Centre of Excellence on Partnership with patients and the public, Montreal, Canada

8: Faculty of Medicine, Université de Montréal, Montreal, Canada

Keywords: patient and family engagement, patient safety, healthcare-associated infections, prevention and control, Q methodology

Word count: 4343

For peer review only

ABSTRACT (250 words)

Introduction

Healthcare-associated infections are an important patient safety concern, especially in the context of the COVID-19 pandemic. Infection prevention and control implemented in healthcare settings are largely focused on the practices of healthcare professionals. Patient and family engagement is also recognized as an important patient safety strategy. The extent to which patients and families can be engaged, their specific roles, and the strategies that support their engagement in infection prevention remain unclear. The overarching objective of the proposed study is to explore how patients and families can effectively be engaged in infection prevention by developing a consensus framework with key stakeholders.

Design and Methods

The proposed study is based on a cross-sectional exploratory study at one of the largest university hospitals in North America (Montreal, Canada). The targeted population is all healthcare professionals, managers, and other non-clinical staff members who work on clinical units, and the in-patients and their families. The study is based on Q methodology that takes advantage of both quantitative and qualitative methods to identify the consensus among the various stakeholders. This exploratory Q research approach will provide a structured way to elicit the stakeholders' perspectives on patient and family engagement in infection prevention.

Ethics and Dissemination

The Research Ethics Board approved this study. The research team plans to disseminate the findings through different channels of communication targeting healthcare professionals, managers in healthcare settings, and patients and family caregivers. The findings will also be disseminated through peer-reviewed journals in healthcare management and in quality and safety improvement.

For peer review only - http://bmjopen.bmj.com/site/about/guidelines.xhtml

Strengths and Limitations of the Study

- The study is based on Q methodology, which is relevant for identifying the consensus among stakeholders for a set of strategies and actions to be implemented in healthcare settings.
- The proposed Q study takes advantage of mixed research methods to understand the perspectives of different key stakeholders on patient and family engagement in infection prevention.
- The study will be conducted with key stakeholders who play a role in infection prevention and control (patients, family caregivers, healthcare professionals, managers, and other non-clinical staff members).
- The study will unfold within a single healthcare organization, which may limit the generalizability of the findings.
- This study will be conducted in different clinical units to compare the different contexts of care.

For peer review only

For peer review only - http://bmjopen.bmj.com/site/about/guidelines.xhtml

Introduction

Healthcare-associated infections (HAIs) are the most frequent complications for patients receiving healthcare. The threat requires increased vigilance in healthcare settings. Internationally, 5% to 15% of patients admitted to hospitals acquire an infection (1). In Canada, HAIs remain particularly persistent with more than 200,000 Canadians being infected every year while receiving healthcare (2). HAIs are infections that are acquired in patients in settings where care is delivered, including healthcare settings (e.g., hospitals or long-term facilities) (3). Examples of bacterial HAIs that are commonly found in healthcare settings include methicillin-resistant Staphylococcus aureus (MRSA) infections, vancomycin-resistant enterococci (VRE) infections, and Clostridium difficile (C. difficile) infections (1, 4). These infections have major health-related and financial consequences for health systems, including higher mortality and morbidity rates and increased lengths of stay at hospitals (5-7).

In the current context of the spread of SARS-CoV-2 in the community, with asymptomatic people spreading the virus, and a proportion of infected people being hospitalized, the incidence of HAIs is expected to increase in the coming months and years. One rapid review and meta-analysis showed that 44% of the confirmed cases of COVID-19 were acquired within healthcare settings (8). Another paper highlighted the risk for inpatients with COVID-19 to have HAIs as secondary infections. Moreover, in adult inpatients diagnosed with COVID-19 in Wuhan, China, half of the non-survivors experienced a secondary infection (9). SARS-CoV-2 is highly contagious and can be rapidly spread in healthcare settings if no specific prevention and control measures are implemented and sustained over time. Thus, the involvement and engagement of patients and their families is needed to prevent spread.

However, to date, infection, prevention, and control (IPC) strategies implemented in healthcare settings have largely focused on the practices of healthcare professionals (HCPs), (e.g., nurses, physicians, allied health professionals) and other staff members (e.g., housekeepers, transport attendants, unit managers), without defining how patients and their relatives could be involved to reduce the infections. Patient and family engagement is

For peer review only - http://bmjopen.bmj.com/site/about/guidelines.xhtml

widely recognized as a promising strategy for improving the quality of healthcare and patient safety (10-13). In recent years, several studies have examined patient engagement in IPC (14-17). In the literature, a consensus has been expressed that patients, family caregivers, and health professionals should play joint roles in preventing HAIs. Nevertheless, the extent to which patients and families can effectively be engaged and their specific roles and responsibilities in IPC remain unclear (18, 19). The evidence suggests that patients may feel anxious or uncomfortable about asking about or getting involved in their safety (20). Studies have also reported that health professionals often acknowledge patients as being vulnerable though not necessarily co-responsible for preventing infection transmission (18). Finally, few studies have identified existing strategies to engage patients and family caregivers in IPC (18), though developing targeted strategies to enable patient and family engagement might contribute to enhance IPC activities.

In line with the recommendations of the World Health Organization (21) and the action plan of the Quebec Ministry of Health and Social Services (22), a Montreal university hospital implemented an interdisciplinary organizational auditing program that aims to reduce HAIs. This nurse-led program, implemented in 2016 in several clinical units focuses on increasing the compliance of: a) hand hygiene, b) appropriate glove use, c) appropriate personal protective equipment use, and d) proper cleaning and disinfection of environment and equipment. To achieve these objectives, four main strategies were implemented: 1) staff training on IPC, IPC tools, and process improvements, 2) audits of hand hygiene, 3) glove use and disinfection of equipment, and 4) communication reinforcement on IPC among staff using huddles (23). Despite improvements in nosocomial infection rates (C. difficile decreased from 13 to 6.8 per 10.000 patient days, MRSA rate decreased from 26 to 12 per 10.000 patient days, and VRE rate decreased from 8 to 4.5 per 10.0000 patient days) and hand hygiene compliance among staff from 37% to 67% (23), audits on clinical units showed that hand hygiene rates among patients and visitors remained low (under 50%). The strategies that were implemented specifically targeted the practices of healthcare professionals (23), without defining how patients and family caregivers could be involved to reduce the infections**.**

For peer review only - http://bmjopen.bmj.com/site/about/guidelines.xhtml

During the pandemic, healthcare organizations have implemented new measures that target patients and family caregivers visiting patients. Some of these measures have increased patient and family responsibilities in infection prevention (e.g., routine hand hygiene, systematic wearing of masks), while other measures have limited the family caregivers' essential roles as partners (e.g., prohibition or limitation of non-essential visits) (24, 25). These restrictive policies were enacted quickly as the pandemic emerged, without input from all stakeholders (including patients, family caregivers, clinical and non-clinical staff members) who play a major role in IPC. Thus, creating a consensus framework will help to define the patients' and family caregivers' roles and responsibilities in IPC during a pandemic and identify the strategies for promoting and facilitating their engagement in this important area of patient safety.

Aims and Objectives

To address the gap, the overarching objective of the study is to explore how patients and families can effectively be engaged in IPC in the context of the COVID-19 pandemic. Specifically, the study is based on the following objectives:

Objective 1: to measure and understand patients' and families' preferences regarding their engagement in IPC.

Objective 2: to measure and understand the preferences of clinical and non-clinical staff on patient and family engagement in IPC.

Objective 3: to identify a consensus framework for the roles and responsibilities of patients and families in IPC and strategies to facilitate their engagement.

Methods and Analysis

Project Design and Research Approach

The proposed study uses a cross-sectional exploratory approach. The targeted population is comprised of all clinical and non-clinical staff members who work in clinical units of the participating healthcare organization that have implemented the interdisciplinary organizational IPC program, and patients hospitalized in these units and their families or other caregivers. The study is based on the Q methodology (26), an exploratory research approach that provides a structured way to elicit the stakeholders' perspectives on the issue

For peer review only - http://bmjopen.bmj.com/site/about/guidelines.xhtml

by using both qualitative and quantitative methods. It offers an innovative alternative to traditional methods for statistically analyzing a variety of perspectives that can lead to the development of a consensus on a set of actions, interventions, or policies for implementation (26, 27).

Project Setting and Stages

The research project will take place at an academic health network that is one of the most modern and largest bilingual university networks in North America (2019-2020: more than 36,000 admissions, around 30,000 surgeries, more than 5,000 nurses and patient attendants, 1,300 physicians and around 4,000 other professionals). The healthcare organization provides tertiary and quaternary care to the population of Montreal and Quebec. The project will be divided into three stages that have been identified as feasible by key stakeholders at the participating healthcare organization.

The Q study is divided into three successive stages (Figure 1). The different sub-sections in the methods section will be presented for each stage successively.

Insert Figure 1

Conceptual Framework

The study relies on a theoretical framework adapted from Pomey et al. (28) on patient and citizen engagement in healthcare systems and on the work of Arnstein (29) on citizen participation in public policy (Figure 2). The framework describes the spectrum of patient and family engagement in infection prevention from paternalism to partnership (co-responsibility). The higher level of engagement involves supporting a shared responsibility among patients, families, and clinical and non-clinical staff members towards infection prevention. At the extreme opposite of the spectrum, mechanisms can be implemented to ensure that patients and families comply with infection prevention measures or are informed about infection prevention practices in hospital settings.

Insert Figure 2

For peer review only - http://bmjopen.bmj.com/site/about/guidelines.xhtml

Stage 1– Elaboration of a set of statements with key experts (fall 2020 - winter 2021)

Objective. The preparatory phase of the study will consist of elaborating on a sample of statements (Q set) on patient and family engagement in IPC that will be used to develop the Q-survey comprising around 40 items that will be administered to participants in the second stage (pilot Q study).

Setting and Participants

The participants will be comprised of key stakeholders involved in the interdisciplinary organizational program focusing on IPC and managers and coordinators responsible for patient and family engagement at the participating healthcare organization. Stakeholders involved in IPC will be recruited through the steering committee of an IPC interdisciplinary organizational program responsible for reflecting, assessing, and orientating appropriate practices in IPC. The IPC program steering committee brings together healthcare professionals (including clinical managers), patients as partners and members from different departments who have a key role regarding IPC (i.e., nursing, medical affairs, infection control, housekeeping, and quality). The IPC program has been ongoing since 2016 and more than 30 units have implemented the strategies targeting improvement in IPC practices within the organization. The principal investigator will present the study during one monthly meeting of the steering committee and members will be invited to participate in the focus group.

In addition, managers and coordinators of patient and family engagement will be recruited through the quality department that is responsible for implementing patient engagement practices within the organization. The participants will be identified with the help of the quality department of the healthcare facility and invited to participate in a focus group by email.

We decided to conduct two separate focus groups, as potential participants represent two groups who might have different viewpoints; the members of the IPC steering committee are interested in IPC measures and strategies and the managers and coordinators of patient

For peer review only - http://bmjopen.bmj.com/site/about/guidelines.xhtml

and family engagement are interested in strategies that foster patient participation in the healthcare facility. We seek to recruit a total of 10 to 12 participants and plan to organize a focus group with each group of stakeholders (five experts per group: members of the steering committee on IPC and managers/coordinators of patient engagement). We adopted a strategic approach to participant recruitment as it is recognized in Q methodology (30); we seek to recruit participants who may have various viewpoints to express that matters in relation to IPC and patient and family engagement (healthcare professionals, other staff members, managers/coordinators, patients as partners). We think that recruiting 10 to 12 participants will ensure this diversity in viewpoints at this stage.

Data Collection and Analysis

Statements on patient and family engagement in IPC will be drawn from two sources. First, a literature review of scientific papers and gray literature will be conducted to highlight key findings in the area of patient engagement in IPC, including the specific context of the COVID-19 pandemic. Second, the qualitative data derived from two focus groups conducted with key experts in IPC and patient and family engagement will be used. Data from these two sources will be integrated to draw a list of approximately 40 statements that represent various perspectives on patient and family engagement in IPC.

Stage 2 – Pilot Q study in one clinical unit (spring and summer 2021)

Objective. This stage will help to measure and understand the preferences of patients, family caregivers, and staff members on patient and family engagement in IPC (Objectives 1 and 2).

This stage will help to identify different groups of perspectives regarding the roles of patients and their families in IPC and the strategies/actions that can be implemented to enhance the preferred roles.

Setting and Participants

This stage will be conducted on one of the clinical units (adult surgery) that has implemented the interdisciplinary program focusing on IPC. This unit has been chosen for

For peer review only - http://bmjopen.bmj.com/site/about/guidelines.xhtml

two reasons: (1) the unit has shown low hand hygiene compliance rates from patients and visitors before the pandemic (37% in 2019) and (2) patients hospitalized in this unit are immunosuppressed and they need greater protection from infections. The unit has been identified and contacted with the help of the nursing department of the participating healthcare facility. The targeted total sample will be 30 participants: 15 members of the clinical unit (health professionals, managers, and other non-clinical staff members) and 15 patients or family caregivers. The recruitment of patients, family caregivers, and staff members will be carried out by the nurse manager and the assistant nurse manager of the unit.

Inclusion criteria. All staff currently working in the clinical unit (HCPs, managers, and other staff members) and who are willing to participate will be included in the study. Among HCPs, we seek to recruit surgeons, nurses, nurse/assistant nurse managers, physiotherapists, and beneficiary attendants. Among other staff members, we seek to recruit housekeepers, clinical managers, unit receptionists. Patients who have been hospitalized or relatives whose loved ones have been hospitalized on these units will be considered for inclusion if they meet the following conditions: (1) 18 years or older; (2) have been hospitalized within the two months before data collection to limit memory biases ; (3) speak and read French or English; (4) have no serious cognitive or speech impairments, or any other conditions that would limit their ability to provide informed consent, and (5) have access to the Internet.

Data Collection

In the pilot Q study, data collection will be based on two methods used in Q methodology (Q surveys and interviews), which will be done remotely because of the COVID-19 pandemic.

All participants will be invited to complete the Q online survey developed in Stage 1. The Q survey will consist of socio-demographic information questions and around 40 statements that participants will have to sort and rank according to a two-step sorting and ranking process specific to the Q methodology. This first step will consist of sorting each

For peer review only - http://bmjopen.bmj.com/site/about/guidelines.xhtml

statement into three categories (agree, neutral, or disagree), and then a second step will ask participants to prioritize statements by ranking them in five categories (-2 completely disagree, -1 disagree, 0 neutral, +1 agree, +2 completely agree). A forced-choice distribution of statements will allow the participants to add a limited and fixed number of statements for each category (4 items in +2/-2, 8 items in +1/-1, 16 items in 0). The Q survey will be hosted on a specific platform (QsorTouch) that has been developed for Q methodologist researchers to easily and securely administrate Q online surveys.

Post-sorting/ranking interviews will be conducted with participants after they have completed the survey and after the researcher has reviewed each participant's Q sort (ranking of statements). As part of the interviews, participants will be asked to explain the rationale of ranking statements in the most extreme scales (i.e., - 2 or +2) and some of the statements ranked in the other scales (0 and -1/+1). Phone interviews will last approximately 30 min and will be audio-recorded.

Data Analysis

Quantitative data from Q surveys. At stage 2, data analysis will consist of comparing and grouping by similarity all of the Q sorts that are collected. The number of Q sorts will be equal to the number of participants (n=30). A by-person factor analysis will be undertaken to measure the intercorrelation of all of the gathered Q sorts to identify a group of persons who ranked the order of statements in a very similar fashion (Q factors) (30). A factor in Q methodology represents a group of persons who share a similar perspective or viewpoint about patient and family engagement in IPC. Grouping, comparing, and summarizing the Q sorts will be performed using data-reduction techniques, including principal component analysis (PCA). The analysis will be done using PQMethods software, which is dedicated to analyzing Q data. PCA consists of reducing the data to a few factors. The reduction in PCA will be done according to two steps: extraction and rotation. Extraction consists of summarizing all of the participants' responses into a few representative responses – the Q perspectives. Rotation is used to have a clearer structure of the results, including an understanding of the percentage of variability explained by the Q perspective (30). In this study, an example of a Q perspective could be participants in favor of controlling patients

For peer review only - http://bmjopen.bmj.com/site/about/guidelines.xhtml

and family members' compliance with IPC practices. Factor loadings will be calculated to explain the relationship between each participant and factor (Q perspective). Z scores will also be examined to "give more precision about how strongly engaged each perspective is with each item" (26).

Qualitative data from interviews. Transcriptions from the interviews will be analyzed using QDA Miner. A thematic analysis will be performed based on our conceptual framework (Figure 2) and following Miles and Huberman methods for qualitative data analysis (Miles & Huberman, 2014).

Integration of quantitative and qualitative data. The interpretation of the results will be based on both quantitative data and qualitative data. Each factor will be described in detail and given a meaningful label to indicate what similar perspectives are about; for example: "controlling patients and family members' IPC practices" or "supporting shared responsibility among patients, family members, and staff members for IPC." Each perspective will reveal and describe the preferences of participants regarding patient and family engagement in IPC. The in-depth qualitative data will allow for an understanding of the rationale of each perspective.

Stage 3 – Validation in four other clinical units (fall 2021- winter 2022)

Objective. This stage of the study is designed to validate the pilot Q findings from a larger sample of clinical units. Since the pilot Q study is based on a small sample, it will not be possible to generalize the findings to a larger population (e.g., other clinical units). This can be done by combining a Q study methodology with an R methodology (traditional survey method) (31).

Setting and Participants

The four participating units at the MUHC will be randomly selected. All staff members (clinical and non-clinical) and patients and family caregivers will be invited to participate in the study. All potential participants will be given a package containing a letter of

For peer review only - http://bmjopen.bmj.com/site/about/guidelines.xhtml

introduction describing the study, a demographic survey, and a link to the survey that will be administrated online.

In Stage 3, the targeted survey sample of participants is 200 participants, equally distributed among the four clinical units that will be randomly selected: 70 patients, 30 family caregivers, 70 HCPs (including managers), 30 non-clinical staff members (housekeepers, transport attendants, etc.). The inclusion criteria for the patients and family caregivers will be the same as for Stage 2.

Data Collection

Creation and administration of a traditional survey. To create a traditional survey, several highly and weakly ranked distinctive statements from each Q perspective measured at Stage 2 will be included and presented as Likert items (a 5-point Likert scale). This technique is the most commonly used for combining Q methodology and survey methods (31, 32). The participants in each clinical unit will be invited to complete the online survey.

Data Analysis

The survey data will be entered in MS-Excel and then exported and analyzed using SPSS software.

Any returned surveys that are missing one or more responses will be excluded from the analysis. For each sample (each clinical unit), we will perform descriptive statistics, calculating mean scores, standard deviations, and the percentage of agreement for each item and the overall scale. High score items shared in the four samples will be identified to develop a consensus framework on the roles of patients and families and strategies that enhance their engagement in IPC. In addition, item scores will be compared across the samples (clinical units) for type of respondents (patients, relatives, HCPs, managers, and other non-clinical staff members), and across different demographic characteristics (age, gender, and level of education). The comparisons will help to identify shared preferences across units, in terms of patient and family roles and strategies to enhance their engagement

For peer review only - http://bmjopen.bmj.com/site/about/guidelines.xhtml

in IPC. The comparisons will also help to show differences between the participants and clinical units in terms of preferences for engaging patients and families in IPC.

Patient and Public Involvement

Patient advisors will be involved in the pilot testing of the online surveys and in the different stages of the study to have their inputs and feedback on the different tools used for data collection and the preliminary results (Stages 1, 2 and 3). One of the collaborators for this study is a patient expert who is a leading member of a reference Canadian center in patient and family engagement.

Discussion

To date, little is known about how patients and families could be effectively engaged in IPC, an important area of patient safety (18, 33, 34). Patient and family engagement in infection prevention remains unclear and is subject to barriers that have been studied previously (18, 35, 36). Moreover, the COVID-19 pandemic has raised important trade-offs between patient safety/IPC practices on one hand and patient and family engagement as essential partners in the other hand (37-39). Developing targeted strategies to overcome the barriers associated with patient engagement is important for patient engagement to become a core component of IPC programs. Engaging patients in IPC can help reduce the burden of HAIs in clinical units and healthcare settings. Because of the complex reactions and barriers regarding patient engagement in patient safety, this study will help define the "optimal" roles of patients in IPC and targeted strategies that are acceptable for stakeholders, including patients, family caregivers, HCPs, managers, and other non-clinical staff members.

The proposed Q study is based on an innovative methodological approach (the Q methodology) that takes advantage of qualitative and quantitative methods to explore the subjectivity of perceptions in a statistical way. Using this methodology is particularly relevant to understand in a structured and clear way the various perspectives on patient and family engagement in IPC. To date, current studies on patient engagement in IPC have

For peer review only - http://bmjopen.bmj.com/site/about/guidelines.xhtml

focused on qualitative methods that provide few insights on consensus strategies among the many actors in the area of patient engagement in patient safety and IPC.

Potential Implications for Healthcare Settings, Patients, and Families

This study can contribute to improving IPC programs that are currently focused on HCP best practices in IPC, including hand hygiene. During this time of pandemic, the importance of engaging all stakeholders in IPC processes is paramount. This research will help develop additional and targeted strategies to better engage patients and their families in patient safety, in the important area of infection prevention. Based on several clinical units, the Q study will also help to elaborate tailor-made strategies of patient engagement in IPC in different clinical contexts.

Ethics and Dissemination

Ethical and Data Protection Considerations

Written consent will be obtained from all participants before each interview. Ethical approval for the current study was obtained from the McGill University Health Center Research Ethics Board approved this study (Patient and Family Engagement / 2020-6404). Electronic data: i.e., information and consent forms, quantitative data from surveys, digital recordings, and interview transcriptions will be password protected and only accessible by members of the research team. Transcription data will be anonymized and audio-recorded interviews destroyed after they are transcribed.

Outputs and Dissemination

This study has been awarded funding by the Social Sciences and Humanities Research Council (SSHRC) as part of the Partnership Engage Grants (PEG) for which we developed a partnership with Healthcare Excellence Canada and a collaboration with the Center of Excellence on Partnership with Patients and the Public (CEPPP). The pandemic has challenged many of the principles of patient partnership in care and the inclusion of family caregivers as essential partners in care, especially with the restrictive visiting policies. In Canada and abroad, the pandemic reminds us of the essential presence of family caregivers to continue to provide moral and physical support particularly to vulnerable patients such

For peer review only - http://bmjopen.bmj.com/site/about/guidelines.xhtml

as older adults and children. In this context, Healthcare Excellence Canada and CEPPP are particularly committed to finding effective ways to better involve patients in IPC and to reintegrate the family presence in healthcare settings. Such a framework will be especially useful for Healthcare Excellence Canada in their 'Better Together' program that focuses on reintegrating family caregivers as essential partners in care. The framework could also be used as a tool designed for health professionals and managers, and patients and family caregivers to help guide and support inclusive engagement initiatives in IPC in the context of a pandemic and beyond.

As part of our partnership and collaboration, various knowledge mobilization activities, including knowledge synthesis, dissemination, and exchange and co-creation with knowledge users and partners will be co-developed. Dissemination activities and approaches will leverage the networks and existing structures of our partners, including Healthcare Excellence Canada and CEPPP, to broadly share the co-created framework to health decision-makers, healthcare professionals, patients, and family caregivers. The research findings will be presented and submitted to international quality and safety management and improvement conferences and journals.

Authors' Contributions

The study concept and design were conceived by NC with substantial feedback from MLT, ABI, and ABR. NC and JP will conduct the data collection and analysis. NC prepared the first draft of the manuscript, and all authors (NC, MLT, ABI, ABR, JP, LB, CF, MPP, and VD) provided edits and critiqued the manuscript for intellectual content. All authors read and approved the final manuscript.

Acknowledgements

We thank the Social Sciences and Humanities Research Council for providing research funding for this study. The authors also thank Glen Wheeler for his contribution to the editing of this manuscript.

For peer review only - http://bmjopen.bmj.com/site/about/guidelines.xhtml

Funding Statement

This study has received funding from the Social and Sciences Humanities Research Council (Government of Canada) through the Partnership Engage Grant – a special initiative for COVID-19 (SSHRC 1008-2020-1062). This study is also supported by a postdoctoral scholarship to Nathalie Clavel from the *Fonds de Recherche du Québec-Santé*. Finally, research funds were provided by the McGill University Health Center and the McGill Nursing Collaborative for Education and Innovation in Patient- and Family-Centred Care (Newton Foundation/McGill Faculty of Medicine) (N/A).

Data-Sharing Statement

The final dataset will be available to study investigators, and to the Research Ethic Boards at the participating site.

Competing Interests' Statement

None declared.

Figures

Figure 1: Study stages

Figure 2: Conceptual framework

For peer review only

References

1. Haque M, Sartelli M, McKimm J, Abu Bakar M. Health care-associated infections - an overview. Infect Drug Resist. 2018;11:2321-33.
2. Health Canada and the Public Health Agency of Canada. Evaluation of Healthcare-Associated Infection Activities at the Public Health Agency of Canada 2012-13 to 2016-17. Ottawa Health Canada; 2018.
3. World Health Organization. Report on the Burden of Endemic Health Care-Associated Infection Worldwide. Geneva World Health Organization 2011.
4. World Health Organization. Drug resistance Geneva World Health Organization; 2021 [Available from: https://www.who.int/drugresistance/AMR_Importance/en/.
5. De Angelis G, Murthy A, Beyersmann J, Harbarth S. Estimating the impact of healthcare-associated infections on length of stay and costs. Clin Microbiol Infect. 2010;16(12):1729-35.
6. Calfee DP. Crisis in Hospital-Acquired, Healthcare-Associated Infections. Annu Rev Med. 2012;63(1):359-71.
7. Tchouaket Nguemeleu E, Beogo I, Sia D, Kilpatrick K, Séguin C, Baillot A, et al. Economic analysis of healthcare-associated infection prevention and control interventions in medical and surgical units: Systematic review using a discounting approach. J Hosp Infect. 2020.
8. Zhou Q, Gao Y, Wang X, Liu R, Du P, Wang X, et al. Nosocomial infections among patients with COVID-19, SARS and MERS: a rapid review and meta-analysis. Ann Transl Med. 2020;8(10):629-.
9. Zhou F, Yu T, Du R, Fan G, Liu Y, Liu Z, et al. Clinical course and risk factors for mortality of adult inpatients with COVID-19 in Wuhan, China: a retrospective cohort study. The Lancet. 2020;395(10229):1054-62.
10. Berger Z, Flickinger TE, Pfoh E, Martinez KA, Dy SM. Promoting engagement by patients and families to reduce adverse events in acute care settings: a systematic review. BMJ Qual Saf. 2014;23(7):548-55.
11. Canadian Patient Safety Institute. Engaging Patients in Patient Safety - a Canadian Guide. Ottawa: Canadian Patient Safety Institute, ; 2017.
12. Kushner C, Davis D. Improving safety: engaging with patients and families makes a difference! Healthc Q. 2014;17 Spec No:41-4.
13. Pomey M-P, Clavel N, Aho-Glele U, Ferré N, Fernandez-McAuley P. How patients view their contribution as partners in the enhancement of patient safety in clinical care Patient Experience Journal 2018;5(1):35-49.
14. Allegranzi B, Conway L, Larson E, Pittet D. Status of the implementation of the World Health Organization multimodal hand hygiene strategy in United States of America health care facilities. Am J Infect Control. 2014;42(3):224-30.
15. Seale H, Chughtai AA, Kaur R, Phillipson L, Novytska Y, Travaglia J. Empowering patients in the hospital as a new approach to reducing the burden of health care-associated infections: The attitudes of hospital health care workers. Am J Infect Control. 2016;44(3):263-8.
16. Pan SC, Tien KL, Hung IC, Lin YJ, Yang YL, Yang MC, et al. Patient empowerment in a hand hygiene program: differing points of view between patients/family members and health care workers in Asian culture. Am J Infect Control. 2013;41(11):979-83.
17. Butenko S, Lockwood C, McArthur A. Patient experiences of partnering with healthcare professionals for hand hygiene compliance: a systematic review. JBI Database System Rev Implement Rep. 2017;15(6):1645-70.

For peer review only - http://bmjopen.bmj.com/site/about/guidelines.xhtml

18. Fernandes Agreli H, Murphy M, Creedon S, Ni Bhuachalla C, O'Brien D, Gould D, et al. Patient involvement in the implementation of infection prevention and control guidelines and associated interventions: a scoping review. BMJ open. 2019;9(3):e025824.
19. Bellissimo-Rodrigues F, Pires D, Zingg W, Pittet D. Role of parents in the promotion of hand hygiene in the paediatric setting: a systematic literature review. J Hosp Infect. 2016;93(2):159-63.
20. Alzyood M, Jackson D, Brooke J, Aveyard H. An integrative review exploring the perceptions of patients and healthcare professionals towards patient involvement in promoting hand hygiene compliance in the hospital setting. J Clin Nurs. 2018;27(7-8):1329-45.
21. World Health Organization. Guidelines on Core Components of Infection Prevention and Control Programmes at the National and Acute Health Care Facility Level. Geneva 2016.
22. Ministère de la santé et des services sociaux. Prévention et contrôle des infections nosocomiales. Plan d'action 2010-2015. Québec Ministère de la santé et des services sociaux,; 2011.
23. Bernard L, Biron A, Briand A, Taha S, Lavoie-Tremblay M. Evaluation of a quality improvement program to prevent healthcare acquired infections in an acute care hospital. Journal of Nursing Education and Practice 2021;11(5):24-31.
24. World Health Organization. Infection prevention and control during health care when COVID-19 is suspected Geneva World Health Organization; 2020.
25. Institut National de la Santé Publique du Québec. Mesures de prévention et contrôle des infections pour les milieux de soins aigus : recommandations intérimaires. Montreal, Quebec: Institut National de la Santé Publique du Québec; 2020.
26. Zabala A, Sandbrook C, Mukherjee N. When and how to use Q methodology to understand perspectives in conservation research. Conserv Biol. 2018;32(5):1185-94.
27. Alderson S, Foy R, Bryant L, Ahmed S, House A. Using Q-methodology to guide the implementation of new healthcare policies. BMJ Qual Saf. 2018;27(9):737-42.
28. Pomey M, Flora L, Karazivan P, Dumez V, Lebel P, Vanier M-C, et al. Le "Montreal Model": enjeux du partenariat relationnel entre patients et professionnels de la santé Sante Publique (Bucur). 2015;HS(S1):41-50.
29. Arnstein SR. A Ladder Of Citizen Participation. J Am Inst Plann. 1969;35(4):216-24.
30. Watts S, Stenner P. Doing Q Methodological Research SAGE, editor. London SAGE; 2012. 238p p.
31. Danielson S. Q Method and Surveys: Three Ways to Combine Q and R. Field Methods. 2009;21(3):219-37.
32. Brown SR. Political Subjectivity: Applications of Q Methodology in Political Science . New Haven Yale University Press; 1980.
33. Bishop AC, Macdonald M. Patient Involvement in Patient Safety: A Qualitative Study of Nursing Staff and Patient Perceptions. Journal of patient safety. 2017;13(2):82-7.
34. Abbasgholizadeh Rahimi S, Zomahoun HTV, Légaré F. Patient Engagement and its Evaluation Tools – Current Challenges and Future Directions; Comment on “Metrics and Evaluation Tools for Patient Engagement in Healthcare Organization- and System-Level Decision-Making: A Systematic Review”. IJHPM. 2019;8(6):378-80.
35. Landers T, Abusalem S, Coty M-B, Bingham J. Patient-centered hand hygiene: The next step in infection prevention. Am J Infect Control. 2012;40(4, Supplement):S11-S7.
36. McGuckin M, Govednik J. Patient empowerment and hand hygiene, 1997-2012. J Hosp Infect. 2013;84(3):191-9.

For peer review only - http://bmjopen.bmj.com/site/about/guidelines.xhtml

37. Canadian Foundation for Healthcare Improvement. Much More than A Visitor: An Executive Summary of Policies in Canadian Acute Care Hospital. Ottawa Canadian Foundation for Healthcare Improvement 2020.
38. Canada Foundation for Healthcare Improvement, Canadian Patient Safety Institute. EVIDENCE BRIEF: Caregivers as Essential Care Partners. Ottawa Canada Foundation for Healthcare Improvement, Canadian Patient Safety Institute; 2020.
39. Canadian Foundation for Healthcare Improvement. BETTER TOGETHER: ReIntegration of Family Caregivers as Essential Partners in Care in a Time of COVID-19 Canada Foundation for Healthcare Improvement; 2020.

For peer review only

For peer review only - http://bmjopen.bmj.com/site/about/guidelines.xhtml

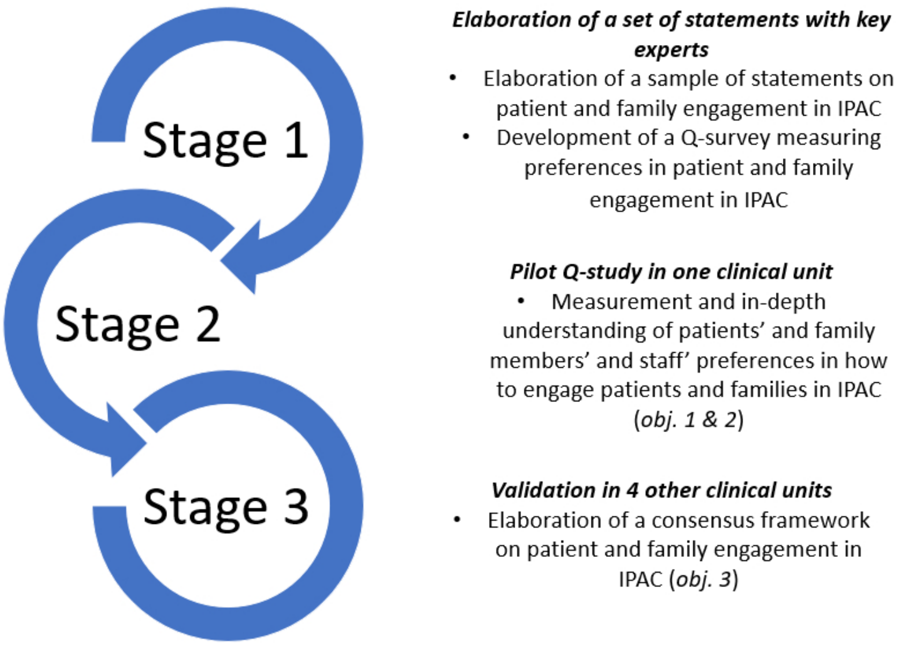

Figure 1_ study stages

385x293mm (57 x 57 DPI)

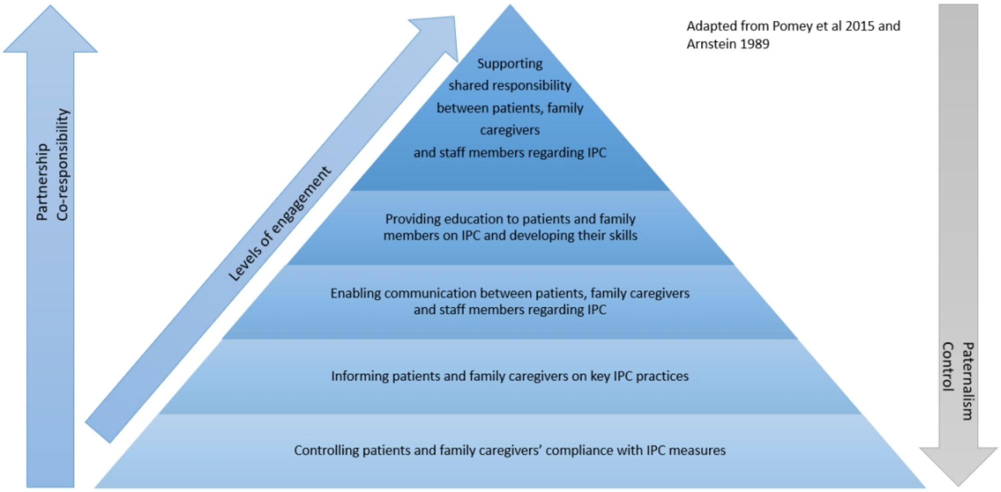

Figure 2_conceptual framework

569x301mm (57 x 57 DPI)

Dimensions and items of the 40-items Q survey

Dimension	Item
**Infection prevention**	• P[1]/FM[2] should receive information on IPV[3] prior to being hospitalize/visit<br>• HP[4] should evaluate P/FM knowledge on IPV before giving them information<br>• HP should communicate information on IPV to P/FM prior to being hospitalize/visit
**Hand hygiene**	• HP are the only ones responsible for complying with HHM[5]<br>• P/FM have responsibilities for complying with HHM<br>• When visiting, FM should routinely practice HHM at key moments<br>• P should routinely practice HHM at key moment<br>• P/FM should ask HP to practice HHM if they notice it has not been done<br>• P/FM don't have the responsibility to check that HP practice HHM<br>• Information about HHM should be available on hospital units<br>• HP should regularly remind P/FM to practice HHM at key moments<br>• PP[6] should educate/encourage P/FM to practice HHM<br>• Hand washing/disinfection stations should be accessible to P/FM<br>• HP should check that P/FM practice HHM at key moments<br>• HP should encourage P/FM to ask HP to wash their hands if they notice it has not been done<br>• Reminders should be available to encourage P/FM to ask HP to wash their hands if they notice it has not been done
**Protective equipment**	• P (outside their room)/FM (entering the hospital) should wear a mask<br>• P (outside their room)/FM (entering the hospital) should not wear a mask<br>• P/FM should ask HP to wear their gloves/mask if they notice it has not been done<br>• P/Fm don't have the responsibility to check that healthcare professionals wear their gloves and mask<br>• Information about mask wearing should be available on hospital units<br>• HP should regularly remind P/FM to wear a mask<br>• PP should educate/encourage P/FM to wear a mask<br>• HP should check that P/FM wear a mask<br>• Masks should be available/accessible on the units for P/FM
**Disinfection**	• P/FM should have the responsibilities to disinfect equipment/immediate surroundings<br>• P/FM do not have the responsibility to disinfect the equipment/immediate surroundings<br>• Information about disinfection should be available to P/FM<br>• HP should regularly remind P/FM to disinfect equipment/immediate surroundings<br>• PP should educate/encourage P/FM to disinfect equipment/immediate surroundings<br>• Disinfecting wipes should be available to P/FM on the hospital units
**Visitors' restrictions**	• FM should replace their visits by virtual visits/phone calls<br>• FM should not limit their visit at the hospital<br>• FM have the role and responsibility to limit their visit, but should not stop visiting<br>• Visits should be prohibited and replaced by virtual visits<br>• Only a limited number of FM should be allowed to visit<br>• All FM should be allowed to visit without exception<br>• HP should encourage FM to do a virtual visit/phone call<br>• HP should ask FM questions prior visiting<br>• FM should sign a document that commits them to respect IPV prior visiting

[1]Patients (P); [2]Family members (PM); [3]Infection prevention measures (IPM); [4]Healthcare professionals (HP); [5]Hand hygiene measure (HHM); [6]Patient partners (PP)

For peer review only - http://bmjopen.bmj.com/site/about/guidelines.xhtml
